# Viral delivery of a peptide-based immunomodulator enhances T cell priming during vaccination

**DOI:** 10.3389/fphar.2022.1029636

**Published:** 2022-12-13

**Authors:** Timothy W. Phares, Jing Huang, Vinayaka Kotraiah, Mary J. Hauser, Arban Domi, Sreenivasa Oruganti, Cecille D. Browne, Peter Buontempo, Marc Mansour, James Pannucci, Moriya Tsuji, Gabriel M. Gutierrez

**Affiliations:** ^1^ Thunder Biotech, Provo, UT, United States; ^2^ The Aaron Diamond AIDS Research Center, New York, NY, United States; ^3^ Department of Medicine, Columbia University Irving Medical Center, New York, NY, United States; ^4^ Leidos Life Sciences, Leidos Inc., Frederick, MD, United States; ^5^ GeoVax Inc., Atlanta, GA, United States; ^6^ Thermo Fisher Scientific, San Diego, CA, United States; ^7^ The MITRE Corporation, McLean, VA, United States; ^8^ MM Scientific Consultants Inc., Halifax, NS, Canada; ^9^ Hibiscus Biotechnology, LLC, Rockville, MD, United States

**Keywords:** vaccine, PD1, immunomodulator, CD8^+^, viral delivery, infectious disease

## Abstract

Modern, subunit-based vaccines have so far failed to induce significant T cell responses, contributing to ineffective vaccination against many pathogens. Importantly, while today’s adjuvants are designed to trigger innate and non-specific immune responses, they fail to directly stimulate the adaptive immune compartment. Programmed cell death 1 (PD-1) partly regulates naïve-to-antigen-specific effector T cell transition and differentiation by suppressing the magnitude of activation. Indeed, we previously reported on a microbial-derived, peptide-based PD-1 checkpoint inhibitor, LD01, which showed potent T cell-stimulating activity when combined with a vaccine. Here we sought to improve the potency of LD01 by designing and testing new LD01 derivatives. Accordingly, we found that a modified version of an 18-amino acid metabolite of LD01, LD10da, improved T cell activation capability in a malaria vaccine model. Specifically, LD10da demonstrates improved antigen-specific CD8^+^ T cell expansion when combined prophylactically with an adenovirus-based malaria vaccine. A single dose of LD10da at the time of vaccination is sufficient to increase antigen-specific CD8^+^ T cell expansion in wild-type mice. Further, we show that LD10 can be encoded and delivered by a Modified Vaccinia Ankara viral vector and can enhance antigen-specific CD8^+^ T cell expansion comparable to that of synthetic peptide administration. Therefore, LD10da represents a promising biologic-based immunomodulator that can be genetically encoded and delivered, along with the antigen, by viral or other nucleic acid vectors to improve the efficacy and delivery of vaccines for ineradicable and emerging infectious diseases.

## Introduction

The development of effective vaccines remains key to eradicating pathogens worldwide. In order to develop successful vaccines, a potent and sustained protective immunity is needed, comprising humoral and cellular immune responses as both are essential for effectively eliminating pathogens. The inability to elicit strong, durable, and protective T cell immunity, particularly CD8^+^ T cell responses, has posed a major obstacle for vaccines and constitutes the primary reason that vaccine development efforts fail, especially for intracellular pathogens (Seder and Hill, 2000). Malaria is a classic example of a disease for which a vaccine is challenging to develop due to lack of T cell immunity.

In an effort to overcome the limitations of current vaccines, adjuvants that enhance T cell immunity are being developed (Counoupas et al., 2017; Halbroth et al., 2018; Thakur et al., 2019). Currently, the majority of adjuvants are designed to generate innate inflammatory danger signals. While these danger signals are essential for innate activation, including antigen presentation and cytokine production, there is limited direct effect on T cells (Petrovsky, 2015; Powell et al., 2015). Thus, novel adjuvants or immunomodulators that directly enhance the expansion and durability of vaccine-induced antigen-specific T cells are needed. Further, novel adjuvants could help decrease the vaccine dosage required to elicit sufficient protective immunity, thereby reducing both toxicity and cost, the latter being a crucial consideration for vaccines intended for developing countries.

Programmed cell death 1 (PD-1) is the most well-characterized checkpoint-inhibitory receptor, and its function is to regulate the threshold, strength, and duration of T cell responses to antigen presentation (Okazaki et al., 2013). PD-1 is rapidly upregulated upon naïve T cell activation, which is required to minimize damage to the host from uncontrolled inflammation during and after infection (Ahn et al., 2018). In non-human primates, immunization with an SIV Gag adenovirus-based vaccine in combination with an anti-PD-1 monoclonal antibody (mAb) significantly elevated peak Gag-specific T cell responses (Finnefrock et al., 2009). Further, we recently showed that antagonizing the PD-1 receptor during prophylactic immunization with an adenovirus-based or radiation-attenuated sporozoite-based malaria vaccine significantly enhanced the number of antigen-specific CD8^+^ T cells (Kotraiah et al., 2020; Phares et al., 2020). These observations suggest that PD-1 modulation may be a critical T cell-focused immunomodulator capable of enhancing T cell expansion and differentiation, resulting in increased numbers and functionality of effector and memory T cells. Similar strategies of combining PD-1 antagonists with a therapeutic vaccine have been used experimentally in cancer therapy to positive effect and are being explored clinically (Massarelli et al., 2019; Verma et al., 2019; Kaumaya et al., 2020; Ott et al., 2020; Peng et al., 2021). Importantly, adjuvant systems such as alum increase the expansion and expression of PD-1 on T cells and may limit their function and their maturation towards effector- and memory-T cell status (MacLeod et al., 2011). Therefore, combining a checkpoint modulator with a traditional adjuvant system may help promote a more balanced immune response in the host. Further, since delivery of vaccine antigens by viral vectors, including Adenoviral and Modified Vaccinia Ankara (MVA) vectors, is a proven strategy for inducing antigen-specific T cell response (Fougeroux and Holst, 2017; Vitelli et al., 2017; Coughlan et al., 2018; Rampling et al., 2018; Folegatti et al., 2020; Förster et al., 2020), combining a checkpoint antagonist with a viral vector-based vaccine may prove advantageous.

While mAb-based checkpoint inhibitors developed to treat cancer can effectively restore immune function, they do not readily lend themselves to the field of infectious disease vaccinology. Due to their long serum half-life, anti-PD-1 mAbs can trigger severe immune-related adverse events (irAEs) and precipitate autoimmune disease (Brahmer et al., 2010; Topalian et al., 2012). Thus, administering such mAbs to a healthy population as a prophylactic vaccine adjuvant poses an unacceptable safety risk. Therefore, peptide-based biologics could be a safer alternative modality to Abs, as peptides have a shorter pharmacokinetic profile and thereby reduce the likelihood of irAEs. Further, peptides offer greater formulation and delivery options and rapid synthetic manufacturing (AlDeghaither et al., 2015; Fosgerau and Hoffmann, 2015; Marqus et al., 2017; Borrelli et al., 2018). Specifically, co-delivery of an immunomodulator genetically encoded in a viral vector would greatly reduce the costs of the vaccine’s formulation. Therefore, we aimed to explore a peptide-based immunomodulator that has potentially greater efficacy in a malaria vaccine model and to demonstrate its delivery by a viral vector platform.

In the current study, we report on LD10, an active, 18-amino acid derivative of our previously reported peptide (Phares et al., 2020). *In vitro*, LD10 demonstrated greater potency at impairing PD-1 receptor signaling relative to LD01. Further, when combined prophylactically with an adenovirus-based malaria vaccine, LD10 treatment resulted in greater expansion relative to LD01 treatment of antigen-specific, IFN-γ-secreting CD8^+^ T cells. Dosing regimen studies established that a single dose of LD10 at the time of immunization with AdPyCS, a circumsporozoite (CS) protein of *Plasmodium yoelii*, was sufficient to enhance the number of vaccine-induced, antigen-specific T cells *in vivo*. Using humanized mice that mimic the human immune system (HIS) and possess functional human CD8^+^ T cells, we demonstrate LD10-mediated modulation of human T cell responses. Moreover, we show that LD10 can be expressed and secreted by a recombinant MVA vector that enhances antigen-specific CD8^+^ T cell expansion. Collectively, these data establish that LD10 is a potent immunomodulator that enhances T cell responses and supports delivery of peptide-based immunomodulators *via* a viral vector.

## Results

### LD10 reduces programmed cell death 1 receptor signaling in a functional cell-based assay

**FIGURE 1:**
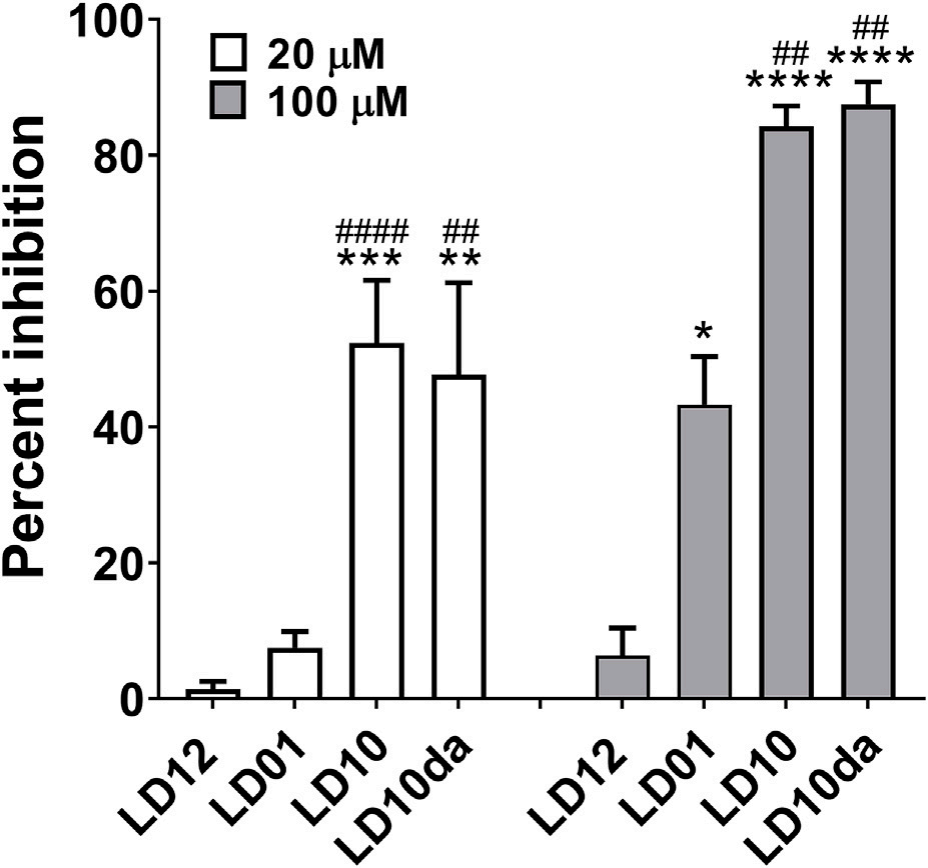
PD-1 receptor signaling is inhibited by LD10. Jurkat cells expressing PD-1 were incubated with LD12, LD01, LD10, or LD10da at 20 μM and 100 µM for 1 h. Programmed death-ligand 1 (PD-L1)-expressing cells were then added to the assay and co‐cultured for 2 h. Chemiluminescence was detected, and the percent of inhibition for the peptides was calculated using the formula described in the *Materials and methods* section. Data represent 3–8 independent experiments and are expressed as the mean ± SEM percent inhibition. Significant differences between LD12 and LD01, LD10, or LD10da at 20 µM and 100 μM, respectively, were determined using a two-tailed unpaired *t*-test and denoted by * (*p* < 0.05), ** (*p* < 0.001) *** (*p* < 0.0005), and **** (*p* < 0.0001). Significant differences between LD01 and LD10 or LD10da at 20 µM and 100 μM, respectively, were determined using a two-tailed unpaired *t*-test and denoted by ^##^ (*p* < 0.01) and ^####^ (*p* < 0.0001).

In order to identify the pharmacophore of LD01, our previously reported 22-amino acid peptide-based PD-1 immunomodulator (Phares et al., 2020), we generated a series of LD01 variants. Analysis of these variants revealed that an 18-mer, in which the first four amino acids of LD01 are deleted, exhibited increased activity in the human PathHunter^®^ PD-1 Signaling Bioassay (Figure 1); we named this 18-mer LD10. In addition, pharmacokinetic analysis *via* liquid chromatography tandem mass spectrometry (LC-MS/MS) of plasma from naïve mice treated intravenously (IV) with a single 200 µg dose of LD01 revealed that the intact peptide circulated for less than 5 min, with metabolites of LD01 detected up to 120 min after administration (Phares et al., 2020). A major LD01 metabolite that was identified in the mouse plasma was the 18-mer LD10 we designed and tested in Figure 1.

These observations prompted us to investigate whether LD10 represented the active metabolite of LD01 *in vivo*. The ability of LD01 and LD10 to demonstrate inhibition in the human PathHunter^®^ PD-1 Signaling Bioassay are shown in Figure 1. In addition to LD01 and LD10, we also tested LD12, a derivative of LD01 that is mutated to severely diminish activity in the PD-1 Signaling Bioassay (Phares et al., 2020) and considered a negative control peptide. LD10da, in which the first amino acid of LD10 was modified to a D-amino acid and the C-terminus was capped with an amide group to protect against terminal degradation by serum proteases was also tested. Peptides were tested at 20 μM and 100 µM. Note, the IC_50_ of a positive control anti-PD1 antibody ran in parallel with the peptides was ∼80 nM. As reported (Phares et al., 2020), incubation with 20 µM LD01 showed minimal inhibition (∼8%) of PD-1 signaling (Figure 1). In contrast, treatment of cells with 20 µM LD10 or LD10da resulted in a mean inhibition of ∼52% and ∼48%, respectively (Figure 1). Moreover, while LD01 resulted in a mean inhibition of ∼43% at 100 μM, LD10 and LD10da each yielded a ∼2-fold greater reduction in PD-1 signaling, with mean inhibitions of ∼84% and ∼88%, respectively (Figure 1). These results indicate that LD10 is an active metabolite of LD01 and that terminal modifications in LD10da appear to have no impact on the activity in the PD-1 Signaling Bioassay. Further, the data indicate that LD10 and LD10da have greater potency relative to LD01 at impairing PD-1 receptor signaling in the current functional cell-based assay.

### LD10da enhances antigen-specific CD8^+^ T cell expansion following adenovirus-based vaccination

**FIGURE 2:**
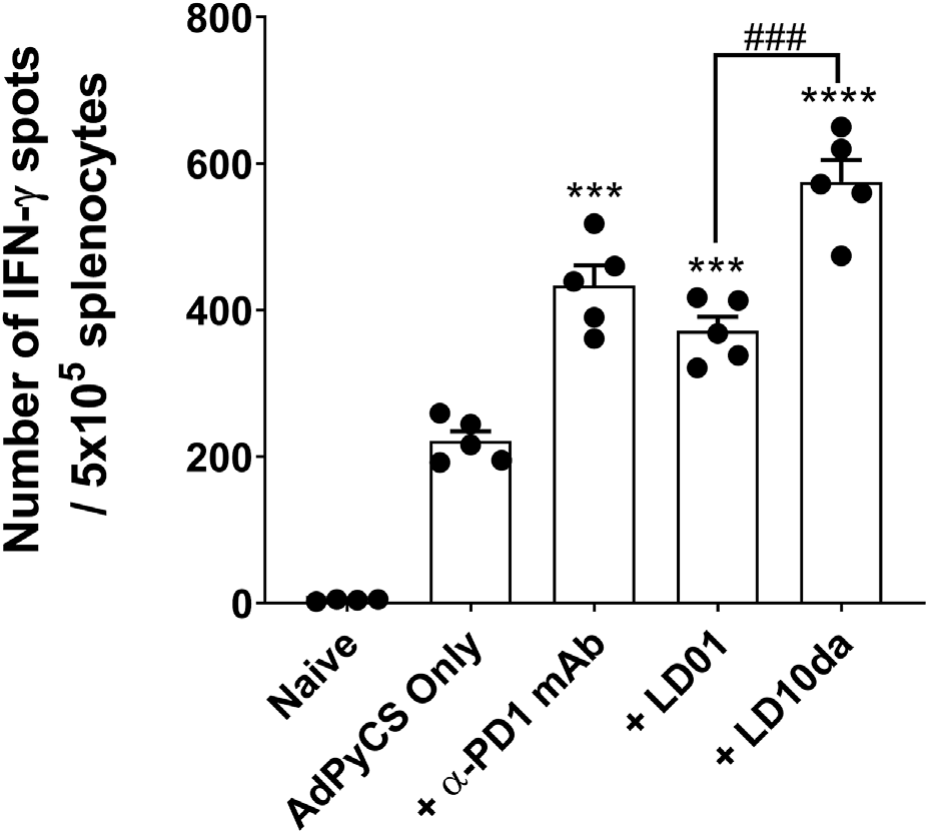
LD10da increases antigen-specific CD8^+^ T cell numbers following AdPyCS vaccination. At D12 post-AdPyCS immunization, immunogenicity was assessed by measuring the number of splenic PyCS-specific, IFN-γ-secreting CD8^+^ T cells using the ELISpot assay after stimulation with the H-2kd restricted CD8 epitope SYVPSAEQI. Data are expressed as the mean ± SEM. Data from one of two independent experiments are shown with *n* = 5 per group. Significant differences between AdPyCS alone and treated mice were determined using a two-tailed unpaired *t*-test and denoted by *** (*p* < 0.0005) and **** (*p* < 0.0001). Significant differences between LD01 and LD10da were determined using a two-tailed unpaired *t*-test and denoted by ^###^ (*p* < 0.0005).

Recently, we demonstrated that LD01, when administered in combination with an adenovirus-based or irradiated sporozoite-based prophylactic malaria vaccine, significantly enhances antigen-specific CD8^+^ T cell numbers (Phares et al., 2020). To assess whether LD10 increases antigen-specific CD8^+^ T cell expansion following vaccination, we used as a model vaccine the recombinant replication-defective adenovirus serotype 5 expressing the entire *P. yoelii* circumsporozoite protein (AdPyCS) (Rodrigues et al., 1997; Phares et al., 2020). The D-amino acid substitution at the N-terminus and the addition of an amide group at the C-terminus of LD10 in LD10da had no impact on its activity *in vitro* (Figure 1). Since these modifications also have the potential to reduce degradation from serum exopeptidases, LD10da was pursued in these *in vivo* studies. Firstly, mice were immunized with AdPyCS intramuscularly (IM). Subsequently, they were treated intraperitoneally (IP) with the α-PD-1 mAb, LD01 or LD10da on day 1 (D1), D3, D5, and D7 (Figure 2). The doses of α-PD-1 mAb and peptides delivered were 200 μg and 100 μg per injection per mouse, respectively. The AdPyCS only group was treated with water, the solvent used to dissolve the peptides, as a control. Splenocytes were isolated from the spleen on D12, and the relative number of PyCS-specific, IFN-γ-secreting CD8^+^ T cells was assessed using an ELISpot assay. Of note, the number of spots seen with naïve splenocytes typically does not exceed five (data not shown). As previously reported (Phares et al., 2020), α-PD-1 mAb and LD01 treatment significantly enhanced the number of PyCS-specific, IFN-γ-secreting CD8^+^ T cells by ∼2 and ∼1.7-fold, respectively, relative to that of mice immunized with AdPyCS alone (Figure 2). Likewise, LD10da significantly increased the number of PyCS-specific, IFN-γ-secreting CD8^+^ T cells (Figure 2), with a ∼2.6-fold change compared to that of mice receiving AdPyCS immunization alone. Notably, LD10da treatment resulted in greater expansion of PyCS-specific, IFN-γ-secreting CD8^+^ T cells relative to LD01 treatment, corroborating the proposition that LD10da may have greater immuno-enhancer potency (Figure 1). Taken together, the data indicate that LD10da treatment significantly enhances the expansion of vaccine antigen-specific T cells *in vivo*.

### A single dose of LD10da at the time of vaccination increases antigen-specific CD8^+^ T cell expansion

**FIGURE 3:**
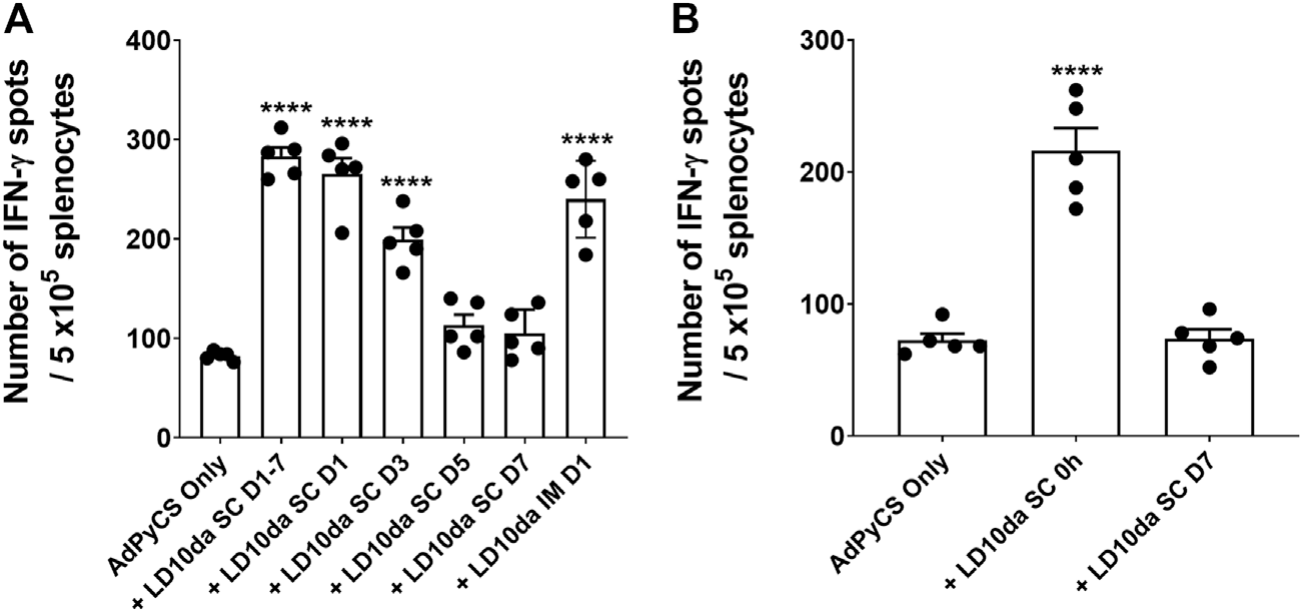
A single dose of LD10da administered concurrently with AdPyCS vaccination increases antigen-specific CD8^+^ T cell numbers. **(A,B)** At D12 post-AdPyCS immunization, immunogenicity was assessed by measuring the number of splenic PyCS-specific, IFN-γ-secreting CD8^+^ T cells using the ELISpot assay after stimulation with the H-2kd restricted CD8 epitope SYVPSAEQI. AdPyCS was administered IM. **(A)** A 100-μg dose of LD10da was given SC either on D1, D3, D5, and D7, or only on D1, D3, D5, or D7 post-immunization. **(B)** A 100-μg dose of LD10da was given SC on D7 or immediately (0 h) post-immunization. Data are expressed as the mean ± SEM. Data from one of two independent experiments are shown with *n* = 5 per group. Significant differences between AdPyCS alone and treated mice were determined using a two-tailed unpaired *t*-test and denoted by **** (*p* < 0.0001).

As described above, the initial dosing regimen for LD10da comprised four administrations, one each on D1, D3, D5, and D7 (Figure 2). To determine whether a single dose of LD10da is sufficient to enhance antigen-specific CD8^+^ T cell numbers following AdPyCS vaccination, mice were treated once on D1, D3, D5, or D7 (Figure 3). Of note, delivering LD10da subcutaneously (SC), relative to IP, does not significantly impact its ability to enhance antigen-specific CD8^+^ T cell expansion (Supplementary Figure S1); therefore, SC delivery was used in these studies. As shown in Figure 3A, treatment with 100 μg LD10da on D1, D3, D5, and D7 significantly increased antigen-specific CD8^+^ T cell numbers relative to mice that were administered AdPyCS alone. Similarly, antigen-specific CD8^+^ T cell expansion was significantly enhanced when LD10da treatment was limited to D1 or D3 (Figure 3A). By contrast, a single administration of LD10da on D5 or D7 following immunization had no effect on antigen-specific CD8^+^ T cell numbers (Figure 3A). Of note, the degree of elevated numbers of antigen-specific CD8^+^ T cells was comparable between SC and IM administration with a single LD10da dose on D1 post-immunization (Figure 3A).

Since a single LD10da dose on D1 following immunization yielded an equivalent increase in antigen-specific CD8^+^ T cell expansion, we next evaluated whether a single LD10da dose administered concurrently with AdPyCS vaccination (0 h) is efficacious (Figure 3B). Indeed, the numbers of antigen-specific CD8^+^ T cells were significantly increased (∼3-fold) when a single LD10da injection was given at the time of vaccination compared to AdPyCS immunization alone (Figure 3B). The absence of increased CD8^+^ T cell expansion with a single administration of LD10da on D7 (Figure 3B) corroborated our previous results (Figure 3A). Taken together, the data indicate that a single SC or IM dose of LD10da at the time of AdPyCS immunization is sufficient to enhance vaccine-induced, antigen-specific T cells *in vivo*, thus indicating its potential as a vaccine adjuvant.

### LD10da promotes vaccine-induced, antigen-specific CD8^+^ T cell expansion in human immune system mice

**FIGURE 4:**
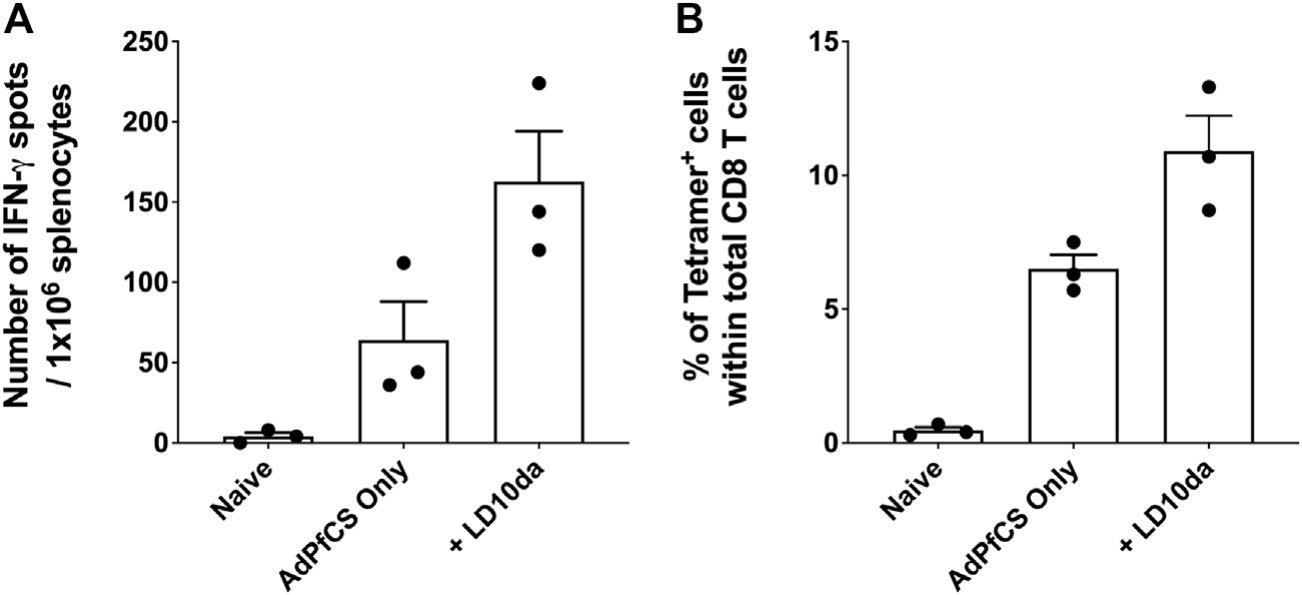
LD10da increases antigen-specific CD8^+^ T cell expansion in AdPfCS-vaccinated HIS mice. At D12 post-AdPfCS immunization, immunogenicity was assessed by measuring the number of splenic PfCS-specific, IFN-γ-secreting CD8^+^ T cells using the ELISpot assay after stimulation with the HLA-A2.1-restricted CD8 epitope YLNKIQNSL **(A)**, or splenocytes from mice were stained directly *ex vivo* for CD3, CD8, and YLNKIQNSL-specific tetramer and analyzed by flow cytometry **(B)**. Data are expressed as mean ± SEM. Data are from a single experiment with *n* = 3 per group.

Reduced PD-1 receptor signaling by LD10da in a human functional cell-based assay suggests that the peptide modulates human T cell responses (Figure 1). To confirm this, we used humanized mice that mimic the HIS and possess functional human CD8^+^ T cells and dendritic cells (Huang et al., 2014; Li et al., 2016; Coelho-Dos-Reis et al., 2020). As detailed in the *Materials and methods* section, HIS mice were generated by engrafting NOD/SCID/IL2Rgamma^null^ (NSG) mice with human hematopoietic stem cells (HSCs) following the transduction of genes encoding several human cytokines and human leukocyte antigen (HLA)-A2.1 by adeno-associated virus serotype 9 vectors (Huang et al., 2014). Flow cytometry analysis confirmed that 85%–95% of the peripheral blood mononuclear cells of HIS mice consist of human CD45^+^ leukocytes, as previously published (Huang et al., 2014). The HIS mice were treated with LD10da at the time of vaccination with a recombinant replication-defective adenovirus expressing the *P. falciparum* circumsporozoite protein (AdPfCS). The dosing regimen for LD10da in the HIS mice was 20 μg on D1, D3, D5, and D7. Of note, the percentages of splenic PD-1^+^ T cells in HIS mice increased following vaccination with AdPfCS (data not shown). As shown in Figure 4A, treatment with LD10da increased antigen-specific CD8^+^ T cell numbers—albeit not significantly—relative to AdPfCS immunization alone without treatment. Similarly, the percentages of splenic HLA-A2.1/YLNKIQNSL-tetramer-specific CD8^+^ T cells were increased following LD10da treatment relative to AdPfCS alone (Figure 4B). This limited experiment provides supporting evidence that LD10da-mediated enhancement of human T cell responses in this *in vivo* model is consistent with its inhibitory activity in the cell-based human PD-1 assay.

### Construction and characterization of an modified vaccinia ankara virus expressing peptide-based immunomodulators

**FIGURE 5:**
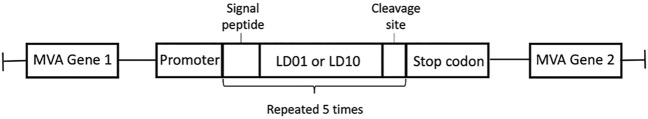
MVA vector construction. Schematic of MVA-5X.LD01 and MVA-5X.LD10 vectors illustrating the design of peptide sequences inserted into the MVA genome between two essential genes under control of an MVA-specific promoter. LD01 and LD10 sequences are preceded by a signal sequence-routing peptide for secretion and followed by a cleavage site to separate duplicated peptides. The secretion signal, peptide sequence, and cleavage site are repeated five times, and transcription is terminated with a stop codon.

Peptide-based therapeutics offer an alternative modality to mAbs and provide a shorter pharmacokinetic profile, thus reducing the likelihood of irAEs. Furthermore, peptide-based biologics can offer a greater number of formulation and delivery options, such as expression *via* a viral vector. To establish whether LD10 could be expressed by a viral vector, we constructed a recombinant MVA virus that encodes five repeats of the LD10 sequence in polycistronic format (MVA-5x.LD10) (Figure 5). In addition to the LD10 construct, a similar recombinant MVA virus expressing five repeats of the LD01 sequence was constructed (MVA-5x.LD01) (Figure 5). To facilitate peptide secretion, a signal sequence was added prior to LD01 or LD10, and a dual cleavage site was added following the sequences in order to facilitate production of the monomer LD01 or LD10 from the polycistronic design.

**FIGURE 6:**
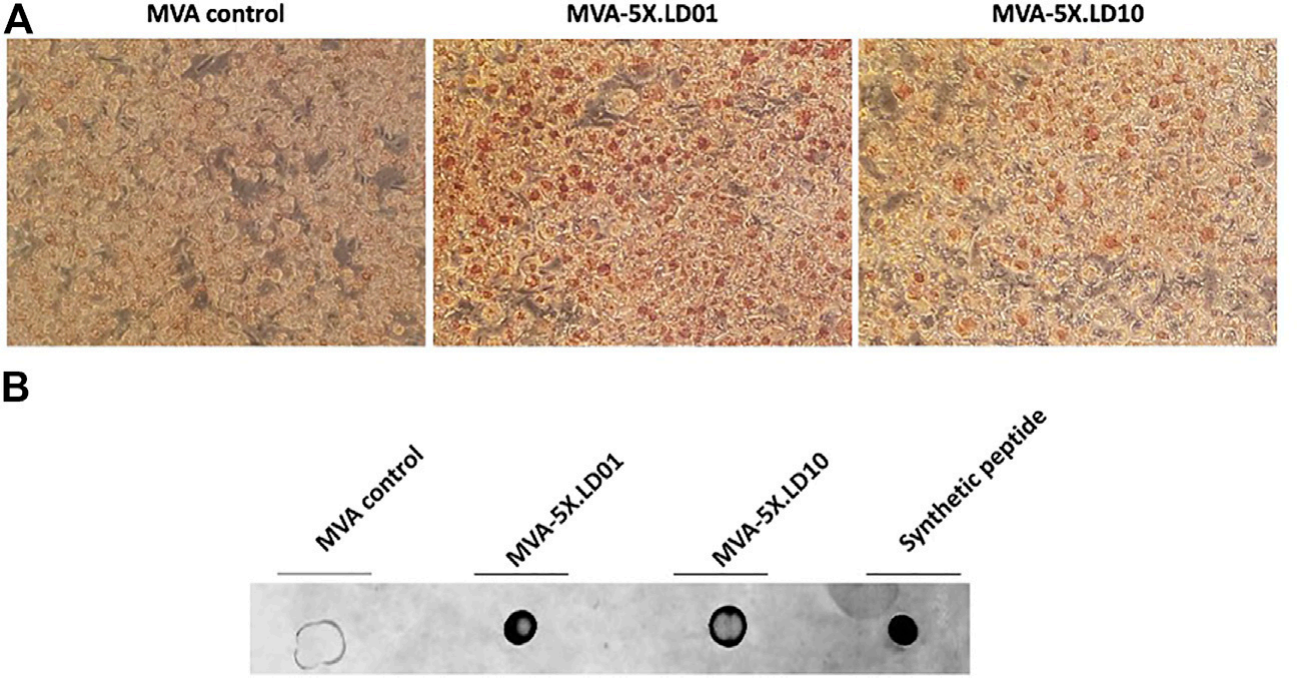
LD01 and LD10 are produced and secreted by MVA-infected cells. **(A)** DF-1 cells were infected with MVA-5X.LD01, MVA-5X.LD10 or parental MVA. Two days post-infection, cells were fixed, permeabilized, and stained with an antibody (Ab) specific to LD01 and LD10. Results show that the peptides are detected intracellularly. Representative images are shown. LD01- and LD10-positive cells are stained brown. Photomicrographs are presented at a magnification of ×20. **(B)** DF-1 cells were infected with MVA-5X.LD01, MVA-5X.LD10, or parental MVA. Two days following infection, supernatant was harvested, concentrated, and dotted onto a membrane along with chemically synthesized peptide (LD01) and probed with an Ab specific for LD01 and LD10.

To determine whether the recombinant MVA vectors express LD01 or LD10, immunohistochemistry was performed on infected cells using a mAb that recognize LD01 and LD10. Of note, cells were fixed and permeabilized with a 50:50 solution of methanol/acetone. Cells infected with the parental MVA vector showed no specific staining (Figure 6A). However, cells infected with either MVA-5X.LD01 or MVA-5X.LD10 vectors showed positive staining (Figure 6A), indicating intracellular expression of the peptides. To confirm that LD01 or LD10 is being secreted by the recombinant MVA vector, a dot blot was performed on infected cell supernatants (see *Materials and methods*). As shown in Figure 6B, the parental MVA vector showed negligible signal. By contrast, both MVA-5X.LD01 and MVA-5X.LD10 vector samples demonstrated positive staining, arguing for secretion of LD01 and LD10. Similarly, LC-MS/MS of the cell supernatants identified LD01 and LD10 fragments (data not shown), corroborating the dot blot results. Taken together, these data strongly suggest that LD01 and LD10 are expressed and secreted by the recombinant MVA vectors.

### Delivery of LD01 or LD10 by modified vaccinia ankara increases antigen-specific CD8^+^ T cell numbers following AdPyCS vaccination

**FIGURE 7:**
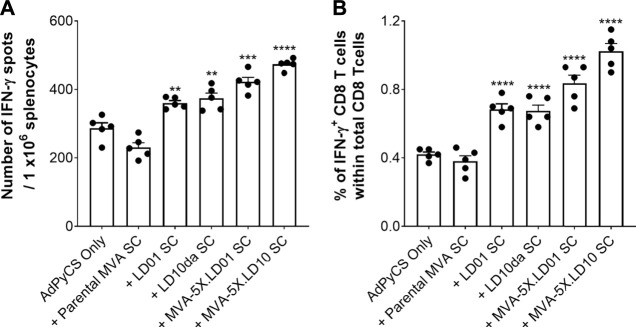
Delivery of LD01 or LD10 *via* a viral vector enhances expansion of vaccine-induced, antigen-specific CD8^+^ T cells. **(A,B)** At D12 post-AdPyCS immunization, immunogenicity was assessed by measuring the number of splenic PyCS-specific, IFN-γ-secreting CD8^+^ T cells using the ELISpot assay **(A)** and flow cytometry **(B)** after stimulation with the H-2kd-restricted CD8 epitope SYVPSAEQI. A 100-μg dose of LD01 or LD10da was given SC immediately following vaccination. For viral vectors, 10^8^ TCID_50_ of MVA-5X.LD01, MVA-5X.LD10, or parental MVA was injected SC following vaccination. Data are expressed as mean ± SEM. Data from one of two independent experiments are shown with *n* = 5 per group. Significant differences between AdPyCS alone and treated mice were determined using a two-tailed unpaired *t*-test and denoted by ** (*p* < 0.001), *** (*p* < 0.0005), and **** (*p* < 0.0001).

Having confirmed that LD01 and LD10 are expressed in and secreted from cells infected with peptide-encoding MVA constructs (Figure 6), we then assessed whether vaccine-induced, AdPyCS-specific CD8^+^ T cell expansion was observed following treatment with MVA-encoding LD01 or LD10. A parental MVA vector was included as a negative control, while synthetic LD01 and LD10da served as positive controls. As shown in Figure 7, treatment with 100 μg of LD01 or LD10da directly following vaccination significantly increased antigen-specific CD8^+^ T cell numbers relative to AdPyCS alone. Similarly, injection of 10^8^ TCID_50_ of MVA-5X.LD01 or MVA-5X.LD10 enhanced antigen-specific CD8^+^ T cell expansion, which contrasted the treatment with the parental MVA vector (Figure 7). Taken together, these *in vivo* results indicate that the delivery of LD01 or LD10 *via* the MVA vector results in immunomodulatory activity that is likely due to their expression *in vivo*. As such, these results corroborate that peptide-based immunomodulators can be successfully delivered by viral vector.

## Discussion

Previously, we showed that peptides are a viable and potentially favorable alternative to mAb-based checkpoint inhibitors for use in infectious disease indications. Improvements in the safety profile, manufacturing costs, and delivery options are among the key advantages of peptide-based biologics. Here we expand on the evidence in favor of peptide-based checkpoint antagonists by generating derivatives of the LD01 peptide, which led to the identification of LD10, a major metabolite whose application concurrent with a model vaccine antigen yields a greater expansion of antigen-specific T cells. We further modified the LD10 peptide by the addition of a C-terminal D-amino acid and an N-terminal amide group (LD10da) with the aim of improving stability. Indeed, we were able to detect the 18-amino acid LD10da peptide for approximately 1 h by LC-MS/MS *via* a pharmacokinetic study in mice treated SC with a single 200 µg dose (data not shown). Therefore, enhanced durability of the LD10 derivative improves the peptide’s immune-modulating properties.

Based on the increased stability of LD10da, we performed a dose-sparing evaluation of the immunomodulator by administering only a single dose at the time of vaccination. Remarkably, this single co-administered dose of LD10da was sufficient to increase antigen-specific CD8^+^ T cell expansion in wild-type and HIS mice. Note that the number of HIS mice used per group was limited due to challenges in breeding NSG mice, availability of HLA-A*0201 matched HSCs, and a long duration (>15 weeks) following HSCs engraftment to ensure human lymphocyte expansion. Therefore, we were unable to obtain another cohort of mice to repeat the experiment. We intend to confirm the HIS mice results in future studies; therefore, the results of this experiment should be interrupted with caution. While the 2-3 fold increase in antigen-specific CD8^+^ T cell expansion following a single dose of LD10da demonstrates a targeted biological effect, whether this improves vaccine efficacy following parasite challenge has yet to be determined and are warranted future studies.

The rationale underlying the remarkably rapid peptide activation of the T cell compartment may be the rapid upregulation of PD-1 *in vivo* upon activation of naïve CD8 T cells. This upregulation can occur 24 h after lymphocytic choriomeningitis virus (LCMV) infection and in less than 4 h after viral peptide injection (Ahn et al., 2018). Accordingly, our findings strongly suggest that modulation of PD-1 signaling does indeed occur shortly after vaccination, and LD10da likely acts on the early activated T cells. Further, it is possible that LD10da binds to T cells constitutively expressing PD-1, as is the case for regulatory CD4 T cells (Tregs) (Francisco et al., 2010). In fact, the PD-1:PD-L1 (programmed death-ligand 1) axis has been shown to play a role in regulating Treg development, expansion, and function (Cai et al., 2019; Lin et al., 2019). For example, PD-L1-deficient antigen-presenting cells only minimally converted naïve CD4 T cells to Tregs, indicating an essential role of PD-1:PD-L1 engagement for Treg induction (Francisco et al., 2009). Further, Francisco et al. (2009) reported that PD-L1 enhances and sustains FOXP3 expression and the suppressive function of Tregs. Similarly, it was shown that dendritic cells overexpressing PD-L1 when co-cultured with naïve CD4 T cells promoted Treg generation, with PD-L1 blockade resulting in reduced Treg expansion (Lin et al., 2019). Thus, it is possible that LD10da binds to the basal PD-1 on Tregs and thereby decreases expansion or inhibitory function, allowing for a greater number of vaccine-induced, antigen-specific CD8^+^ T cells. While we have yet to assess the Treg responses following AdPyCS vaccination in the absence or presence of our peptide-based immunomodulators, we recently reported that LD01 treatment of mice infected with a lethal malaria strain resulted in survival that was associated with lower numbers of FOXP3^+^Tbet^+^CD4^+^ Tregs (Phares et al., 2020). Tregs also constitutively express cytotoxic T-lymphocyte antigen 4 (CTLA4), which is critical for their suppressive abilities. In this regard, we have obtained preliminary data showing that LD10da and LD01 antagonize a novel allosteric site that is shared by the CD28 family of checkpoint receptors (data not shown), of which CTLA4 is a member. Therefore, these peptide-based immunomodulators may be modulating Treg activity by disrupting both PD-1 and CTLA4 signaling.

In addition to modifying T cell responses there is a growing body of literature demonstrating PD-1 expression on myeloid cells mediates cellular dysfunction (Huang et al., 2009; Strauss et al., 2020; Phares et al., 2021). For example, we recently showed in a cecal-ligation and puncture-induced murine polymicrobial sepsis model that LD01 treatment alleviated aspects of phagocyte immune dysfunction (Phares et al., 2021) corroborating the pathologic role of PD1 in altering microbial clearance and innate immunity in sepsis (Huang et al., 2009). Further, ablation of myeloid cell-specific PD-1 in a tumor model resulted in reduced tumor accumulation of myeloid-derived suppressor cells and increased T effector memory cell function enhancing overall antitumor protection (Strauss et al., 2020). Interestingly, myeloid cell-specific PD-1 ablation also increased cholesterol (Strauss et al., 2020), a molecule that drives myeloid cell expansion and differentiation and promotes antigen presenting function, which raises the possibility that the improved T cell function is partially due to enhanced antigen presentation in the tumor model. Similarly, in the AdPyCS vaccine model the increased antigen-specific CD8^+^ T cell expansion observed after PD-1 blockade may be a consequence of enhanced antigen presentation by myeloid cells, something we intend to assess in future studies.

While we opted to use the AdPyCS vaccine model to rapidly test many LD01 derivatives, including LD10, we recognize that the dosing regimen of our peptides for traditional vaccines with a prime/boost(s) regimen may differ. In fact, in a therapeutic cancer vaccine model, the timing and order of vaccine and anti-PD-1 treatment have been shown to be crucial in determining optimal CD8^+^ T cell responses and therapeutic outcomes (Verma et al., 2019). Preliminary studies that we conducted with soluble vaccine antigens in a 3-dose regimen showed that the number of antigen-specific T cells generated in mice treated with LD01 following the second and third immunization was greater than the numbers of these cells generated with treatment after each immunization (data not shown). Additionally, in a whole-cell vaccine model, we have obtained data indicating that LD10da treatment following a primary and booster vaccination reduces bacterial challenge burdens in the nasal passage and lungs to a greater degree than did administration of the LD10da only after the primary immunization (data not shown). Accordingly, dosing regimens to modulate PD-1 activity are likely dependent on an optimized schedule of priming and boosting relative to antigen stimulation, a hypothesis that we intend to test in future studies.

Given that the more stable LD10 peptide has been proven to be a potent immunomodulator in a vaccine formulation, we aimed to establish proof of a potential delivery platform for future vaccine development efforts by encoding the peptide in the MVA platform. Following IM infection of mice with recombinant MVA-expressing green fluorescent protein (rMVA-GFP), GFP^+^ cells were identified as myocytes and interdigitating cells having a macrophage or dendritic cell morphology (Altenburg et al., 2017). Further, GFP^+^ cells were detected in the draining lymph nodes as well as systemically in white blood cells and splenocytes (Altenburg et al., 2017). Additionally, *in vitro* human peripheral blood mononuclear cell (PBMC) assays and *ex vivo* mouse lung explants showed that rMVA-GFP predominately infects dendritic cells (Altenburg et al., 2017). These data suggest that our peptide-based immunomodulators are expressed by MVA at the site of injection and/or within the lymphoid tissues. Of note, MVA is also detected in lymphoid organs following SC injection (Ramirez et al., 2003). Expression of LD10 or LD01 within the lymphoid tissues is ideal given that these tissues are the primary site of T cell activation, differentiation, and expansion. Moreover, our peptides are believed to be secreted locally during T cell priming because MVA preferentially targets dendritic cells (Altenburg et al., 2017) and because these professional antigen-presenting cells directly interact with or are in close proximity to T cells during immune responses. In future efforts, we plan to identify the specific cell(s) that may be expressing the peptide-based immunomodulators and to specify the duration of expression and circulating levels. These data are important for advancing our understanding of the basis for the immunogenicity of MVA-based vaccines and for informing effective vaccine designs and delivery strategies.

In addition to our reported efforts on MVA-based vaccine development, we are currently developing chimeric antigen receptor (CAR) T cells, which have been genetically engineered to express LD10. Here the secreted LD10 would not only bind to the PD-1 expressed by the CAR T cell, but also potentially engage and relieve dysfunction of endogenous T cells. We have also proceeded with the development of oncolytic viruses that express our peptide-based immunomodulators. With such viruses, which can be engineered to replicate selectively in tumor tissues, LD01 or LD10 would be produced within the infected cell and released into the tumor microenvironment following virus-induced lysis. Moreover, we are developing a dissolving microneedle patch that facilitates a painless means of delivering our peptides into the dermis, which easily accesses the lymphatic system. As microneedle patches are a viable means of circumventing the challenges associated with conventional vaccine delivery, we envision combining our peptide-based immunomodulators with vaccine antigens. In addition to the microneedle patch, we are currently testing other delivery platforms, including a solid-dose implant that is needle-free and delivers the peptides transcutaneously into the dermis. Akin to the microneedle patch, our peptide-based immunomodulators could be combined with vaccine antigens in a single implant. All of the aforementioned efforts may allow us to develop potent T cell-stimulating infectious disease vaccines that can be stably deployed and easily administered in more austere environments.

## Materials and methods

### Ethics statement

All animal experiments were carried out in strict accordance with the Policy on Humane Care and Use of Laboratory Animals of the United States Public Health Service. The protocol was approved by the Institutional Animal Care and Use Committee (IACUC) at The Rockefeller University (Assurance No. A3081-01). Mice were euthanized with CO_2_, with every effort made to minimize suffering. Human fetal liver samples were obtained *via* a non-profit partner (Advanced Bioscience Resources, Alameda, CA, United States). As no information was obtained that would identify the subjects from whom the samples were derived, Institutional Review Board approval for their use was not required, as previously described (Huang et al., 2014).

### Programmed cell death 1:Programmed death-ligand 1 cell-based reporter assay

For the PathHunter^®^ PD‐1 signaling assay (cat# 93-1104C19; Eurofins DiscoverX; Fremont, CA, United States), Jurkat cells expressing PD-1 and SHP1 proteins, each fused to a fragment of DiscoverX’s enzyme fragment complementation (EFC) system, were co‐incubated with ligand‐presenting cells. This led to PD-1 activation and SHP1 recruitment to the PD-1 receptors, bringing together the two EFC fragments and generating a chemiluminescent signal. In this assay, LD01, LD10, LD10da, and LD12 were assessed at 20 µM and 100 μM, whereas anti-PD-1 mAb controls were assessed at 10 different concentrations. In brief, PD‐1-expressing Jurkat cells (20,000 cells/well) were seeded in a total volume of 50 μl into white-walled, 96‐well microplates in assay buffer. Serial dilution of LD01 stock was performed to generate an ×11 sample in assay buffer, and 10 μl of the ×11 test sample was added to PD-1 cells and incubated at 37°C for 1 h U2OS cells expressing PD‐L1 (50 μl, 30,000 cells/well in assay buffer) were then added to the assay. Cells in co‐culture were incubated at room temperature (RT) for 2 h (PD-1 assay). The assay signal was generated using the PathHunter^®^ Bioassay Detection Kit for both assays. Detection reagent 1 (10 μl) was added to the assay and incubated at RT for 15 min. Detection reagent 2 (40 μl) was added to the assay and incubated at RT for 1 h. Microplates were read following signal generation with an EnVision™ plate reader (PerkinElmer, Waltham, MA, United States) for chemiluminescent signal detection. LD01 activity and anti-PD-1 mAb activities were analyzed using the CBIS data analysis suite (ChemInnovation Software, Inc., San Diego, CA, United States). For antagonist mode assays, the percentage of inhibition by the peptides was calculated using the following formula: $percent\;inhibition\;efficacy=100\%\times \left[1\;–\;\;\left(mean\;RLU\;of\;test\;sample\;–\;\;mean\;RLU\;of\;vehicle\;control\right)\;/\;\left(mean\;RLU\;of\;EC80\;control\;–\;mean\;RLU\;of\;vehicle\;control\right)\right].$

### Mice

Female BALB/c mice 6–8 weeks of age were purchased from The Jackson Laboratory (Bar Harbor, ME, United States). NOD/SCID/IL2Rgamma^null^ (NSG) mice exhibiting features of both severe combined immunodeficiency mutations and interleukin (IL)-2 receptor gamma-chain deficiency were also purchased from The Jackson Laboratory and maintained under specific pathogen-free conditions.

### Generation of human immune system-CD8 mice

Recombinant AAV9 (rAAV9) vectors encoding human IL-3, IL-15, GM-CSF, and HLA-A*0201 were constructed as previously described (Huang et al., 2014). Four-week-old NSG mice were transduced with rAAV9 encoding HLA-A*0201 by perithoracic injection and with rAAV9-encoding HLA-A*0201 and AAV9-encoding human IL-3, IL-15, and GM-CSF, by IV injection, as previously described (Huang et al., 2014). Two weeks later, mice were subjected to 150-Gy total body sub-lethal irradiation for myeloablation, and several hours later, each transduced, irradiated mouse was engrafted IV with 1 × 10^5^ HLA-A*0201^+^-matched, CD34^+^ human HSCs. CD34^+^ HSCs among lymphocytes derived from HLA-A*0201^+^ fetal liver samples were isolated using a Human CD34 Positive Selection Kit (STEMCELL Technologies, Vancouver, BC, United States) (Lepus et al., 2009). At 14 weeks post-HSC engraftment, the reconstitution status of human CD45^+^ cells in the blood of HIS-CD8 mice was determined by flow cytometry analysis, as previously described (Huang et al., 2014).

### AdPyCS and AdPfCS vaccines

A recombinant serotype 5 adenovirus that expressed *P. yoelii* circumsporozoite protein (PyCS), AdPyCS, was constructed as previously described (Rodrigues et al., 1997). A recombinant adenovirus serotype 5 (Ad5) expressing a GFP alone in its transgene, AdGFP, was previously constructed (Shiratsuchi et al., 2010). A recombinant Ad5 expressing *P. falciparum* CSP (AdPfCS) was also previously constructed as described (Altenburg et al., 2017). Briefly, a gene encoding a full-length PfCSP was codon-optimized and synthesized, and then inserted into pShuttle-CMV, which was used to make the recombinant AdPfCS. Each BALB/c mouse was immunized IM with 5 × 10^9^ virus particles of AdPyCS, whereas each HIS-CD8 mouse was immunized IM with 1 × 10^10^ virus particles of AdPfCS.

### ELISpot assay and flow cytometry to measure antigen-specific CD8^+^ T cells

As previously described (Li et al., 2016), the relative numbers of splenic PyCS-specific, IFN-γ-secreting CD8^+^ T cells of AdPyCS-immunized mice were determined by ELISpot assay using a mouse IFN-γ ELISpot Kit (Abcam, Cambridge, MA, United States) and a synthetic 9-mer peptide, SYVPSAEQI (Peptide 2.0, Chantilly, VA, United States) corresponding to the immunodominant CD8^+^ T cell epitope within PyCS (Li et al., 2016). In brief, after the collection of splenocytes from mice 12 days after AdPyCS immunization, 5 × 10^5^ splenocytes were placed in each well of the 96-well ELISpot plates pre-coated with IFN-γ Ab and incubated with the SYVPSAEQI peptide at 5 μg/ml for 24 h at 37°C in a CO_2_ incubator. After the ELISpot plates were washed, they were incubated with biotinylated anti-mouse IFN-γ Ab for 2–3 h at RT, followed by incubation with avidin conjugated to horseradish peroxidase for 45 min at RT in the dark. Finally, the spots were developed after the addition of the ELISpot substrate (Abcam).

To identify the number of IFN-γ-secreting CD8^+^ T cells in each well, the mean number of spots (for duplicates) counted in the wells incubated with splenocytes in the presence of the peptide was subtracted by the mean number of spots (for duplicates) counted in the wells that were incubated with splenocytes only. The percentage of antigen-specific IFN-γ^+^ T cells among splenocytes of immunized mice were also determined by intracellular cytokine staining. Briefly, splenocytes harvested from vaccine-immunized animals were stimulated for 6 h by co-culture with 5 μg/ml SYVPSAEQI peptide at 37°C in the presence of Brefeldin A Solution (cat# 420601, BioLegend). Cells were then incubated for 15 min in the presence of TruStain FcX™ PLUS (anti-mouse CD16/32) (cat# 101319, BioLegend) before surface staining for 30 min with α-CD3ε-PE/Cy7 (cat# 100220, BioLegend), α-CD8α-PE/Cy5 (cat# 100710, BioLegend), and α-CD4-AF700 (cat# 100536, BioLegend). Permeabilization was performed using Fixation Buffer (cat# 420801, BioLegend) followed by washing with Intracellular Staining Perm Wash Buffer (cat# 421002, BioLegend) according to the manufacturer’s instructions. Cells were stained intracellularly for 30 min with α-IFN-γ-APC (cat# 505810, BioLegend). Flow cytometry analyses were performed using an LSRII (BD Biosciences, San Jose, CA), and data were analyzed using FlowJo™ v10 (TreeStar, Ashland, OR).

### Staining with HLA-A/0201 tetramer loaded with YLNKIQNSL peptide

An allophycocyanin (APC)-labeled human HLA-A*0201 tetramer loaded with the peptide YLNKIQNSL, corresponding to the PfCSP CD8^+^ T cell epitope (Blum-Tirouvanziam et al., 1995; Bonelo et al., 2000), was provided by the NIH Tetramer Core Facility. Twelve days after immunization of HIS-CD8 mice with AdPfCS, the spleens were harvested, and splenocytes were stained with APC-labeled human HLA-A*0201 tetramer loaded with YLNKIQNSL and PE-labeled anti-human CD8 Ab (BioLegend). The percentage of HLA-A*0201-restricted, PfCSP-specific CD8^+^ T cells among the total human CD8^+^ T cell population was determined using a BD™ LSR II flow cytometer (Franklin Lakes, NJ), as previous performed (Li et al., 2016).

### Modified vaccinia ankara construction and seed stock preparation

Two recombinant MVAs, MVA-5x.LD01 and MVA-5x.LD10, were constructed that encode five repeats of LD01 or LD10 sequences in polycistronic format. A tissue plasminogen activator signal sequence was added prior to LD01 or LD10 to route the peptides for secretion from the cell, and a dual cleavage site composed of the porcine teschovirus-1 2A sequence followed by a furin cleavage peptide were added following the LD sequences to facilitate production of monomer peptides from the polycistronic design. The starting material for recombinant virus production was parental MVA that had been harvested in 1974 before the appearance of Bovine Spongiform Encephalopathy/Transmissible Spongiform Encephalopathy (BSE/TSE) and that had been plaque-purified three times using certified reagents from sources free of BSE. A shuttle vector was used to insert the LD01 or LD10 sequences (U.S. Patent 10,799,581 and EP Patent 3512536) between two essential genes of MVA by means of homologous recombination. The chosen insertion site was previously identified as supporting high expression and insertion stability. Genetic stability of the transgene inserts was confirmed by PCR to ensure the correct size. All inserted sequences were codon-optimized for MVA. Silent mutations were introduced to interrupt homo-polymer sequences (>4G/C and >4A/T), which reduce RNA polymerase errors that possibly lead to frameshift mutations. All vaccine inserts were placed under control of the modified H5 early/late vaccinia promoter. Vectors, Research Seed Virus (RSV), and Research Stocks (RS) were prepared in a dedicated room at GeoVax, with full traceability and complete documentation of all steps using BSE/TSE-free raw materials and, therefore, can be directly used for production of cGMP Master Seed Virus (MSV).

For production of RSV for animal studies, a chicken embryo fibroblast cell line, DF-1 cells (ATCC, CRL-12203), were seeded into sterile tissue culture flasks and infected with MVA-5x.LD01 or MVA-5x.LD10 at an MOI of 0.01. Cells were recovered 3 days post-infection, disrupted by sonication, and bulk harvest material clarified by low-speed centrifugation. The clarified viral harvest was purified twice using sucrose cushion ultracentrifugation. The purified viruses were titrated by limiting dilution in DF1 cells, diluted to 1 × 10^8^ TCID_50_/ml in sterile PBS + 7% sucrose, dispensed into sterile vials, and stored at −80°C. For *in vivo* studies, mice were injected with 50 µl containing 5 × 10^7^ TCID_50_ of MVA into each hind footpad for a total dose of 1 × 10^8^ TCID_50_ MVA per mouse.

### Production of anti-LD01/LD10 mAb

KLH-conjugated LD01 peptide formulated in Sigma Adjuvant System^®^ (cat# S6322) was used to immunize SJL/J mice IM. Following two similar IM boosts at 2-week intervals, the mice were culled, and spleens and lymph nodes were collected. Splenocytes and lymphocytes were isolated and fused to HL-1 mouse myeloma cells and cultured for 13 days. On D13, colonies were manually selected and transferred to selection media. Culture supernatants were screened for specificity by ELISA using plate-coated BSA-conjugated peptides. Supernatants were screened against BSA-conjugated LD01 and LD10 peptides. Two clones (3F11 and 7G10) were selected based on their high binding affinity to both peptides and on the high concentration of supernatant Ab. Monoclonal cultures of these two clones were expanded and the supernatants were used to purify the Abs. Cell suspensions, containing at least 8.0 × 10^7^ cells in two T-75 flasks, were aseptically transferred to 2 ml × 50 ml centrifuge tubes and centrifuged at 1,000 rpm for 5 min. The resulting cell pellet was resuspended in 25 ml of HyClone™ HYQSFMMAB media + 5% FBS and slowly added to a 250-ml bag containing 225 ml of HyClone™ HYQSFMMAB media + 5% FBS. The bag was placed in an incubator set at 5% CO_2_, 37°C for 10–14 days. After 10–14 days of growth, the contents of the 250 ml bag were transferred to a 250 ml centrifuge bottle, and 10 ml of Neutralization Buffer (1 M Tris, 1.5 M NaCl, pH 8.5) was added to it; it was then centrifuged at 8,600 rpm for 10 min using a Sorvall GSA rotor. The supernatant was filtered using a 0.45-μm bottle top filter.

A 5-ml protein A column connected to a FPLC Purification System was washed with 25 ml of ultra-pure water followed by 25 ml of 50 mM TRIS containing 250 mM NaCl (pH 8.0). The filtered supernatant was loaded onto the column at a flow rate of 7 ml/min. The column was further washed with 15 ml of 50 mM TRIS, 250 mM NaCl, pH 8.0. Elution fractions were collected in 15-ml tubes containing 800 μl of Neutralization Buffer (1 M Tris, 1.5 M NaCl, pH 7.4). The Ab was eluted with 20 ml of 50 mM glycine (pH 3.0) and dialyzed against 1–2 L of 1× PBS (pH 7.4; depending on the volume of purified Ab) on a stirrer at 4°C overnight. The dialyzed Ab was sterile-filtered and aliquoted for storage.

### Dot blot assay

DF-1 cells were infected at a multiplicity of infection of 0.5 with parental MVA, MVA-5X.LD01 or MVA-5X.LD10 and 48 h later supernatant was collected. In order to concentrate secreted peptide, supernatant was passed through Pierce C-18 tips (cat# 87782, Thermo Fisher Scientific). Twenty microliters from each sample and 125 ng of synthetic LD01 peptide were spotted onto a PVDF membrane, allowed to dry at room temperature, then blocked with Intercept blocking buffer (cat# 927-70001, LI-COR Biosciences) for 30 min at room temperature. The membrane was incubated overnight at 4°C in primary Ab (Leidos, clone: 7G10) diluted in blocking buffer at 1:1,000. Three washes with PBST (PBS with 0.05% Tween-20) were performed, and the membrane was probed for 1 h with anti-mouse-680RD (1:10,000; cat# A-21058; Invitrogen). The membrane was then washed again and imaged using Odyssey imager.

### Immunocytochemistry assay

DF-1 cells were infected at a multiplicity of infection of 0.5 with parental MVA, MVA-5X.LD01 or MVA-5X.LD10 for 48 h, subsequently cells were fixed in 1:1 methanol:acetone and washed with water. Cells were then probed with a mouse anti-LD01/LD10 Ab (Leidos, clone: 3F11) at room temperature for 1 h. Three washes with water were performed and the cells were stained for 1 h with anti-mouse-IgG-HRP (1:1,000; VWR, cat# 10150-400). The cells were then washed again and developed with AEP substrate kit (cat#ab64252, Abcam). Images of stained cells were captured at ×20 magnification using light microscopy.

### Data analysis

Statistical analyses were performed using Prism (GraphPad Software, Inc., La Jolla, CA, United States). The two-tailed unpaired *t*-test was used to determine differences between groups. Data are expressed as mean ± SEM and *p* < 0.05 was considered statistically significant.

## Data Availability

The full complement of data accumulated for these studies is available upon request.

## Supplementary material

SUPPLEMENTARY FIGURE S1 IP and SC administration of LD10da demonstrate similar increases in antigen-specific CD8^+^ T cell numbers following AdPyCS vaccination. At D12 post-AdPyCS immunization, immunogenicity was assessed by measuring the number of splenic PyCS-specific, IFN-γ-secreting CD8^+^ T cells using the ELISpot assay after stimulation with the H-2kd restricted CD8 epitope SYVPSAEQI. A 100-μg dose of LD10da was given either IP or SC on D1, D3, D5, and D7 post-immunization. Data are expressed as the mean ± SEM fold increase in the number of IFN-γ spots with the levels from AdPyCS alone taken as one. Data from one of two independent experiments are shown with *n* = 5 per group.
