# Supplementary figures and images for: Viral delivery of a peptide-based immunomodulator enhances T cell priming during vaccination
Source: Front Pharmacol. 2022 Dec 13;13:1029636. doi: 10.3389/fphar.2022.1029636 (PMC9792674; doi:10.3389/fphar.2022.1029636)

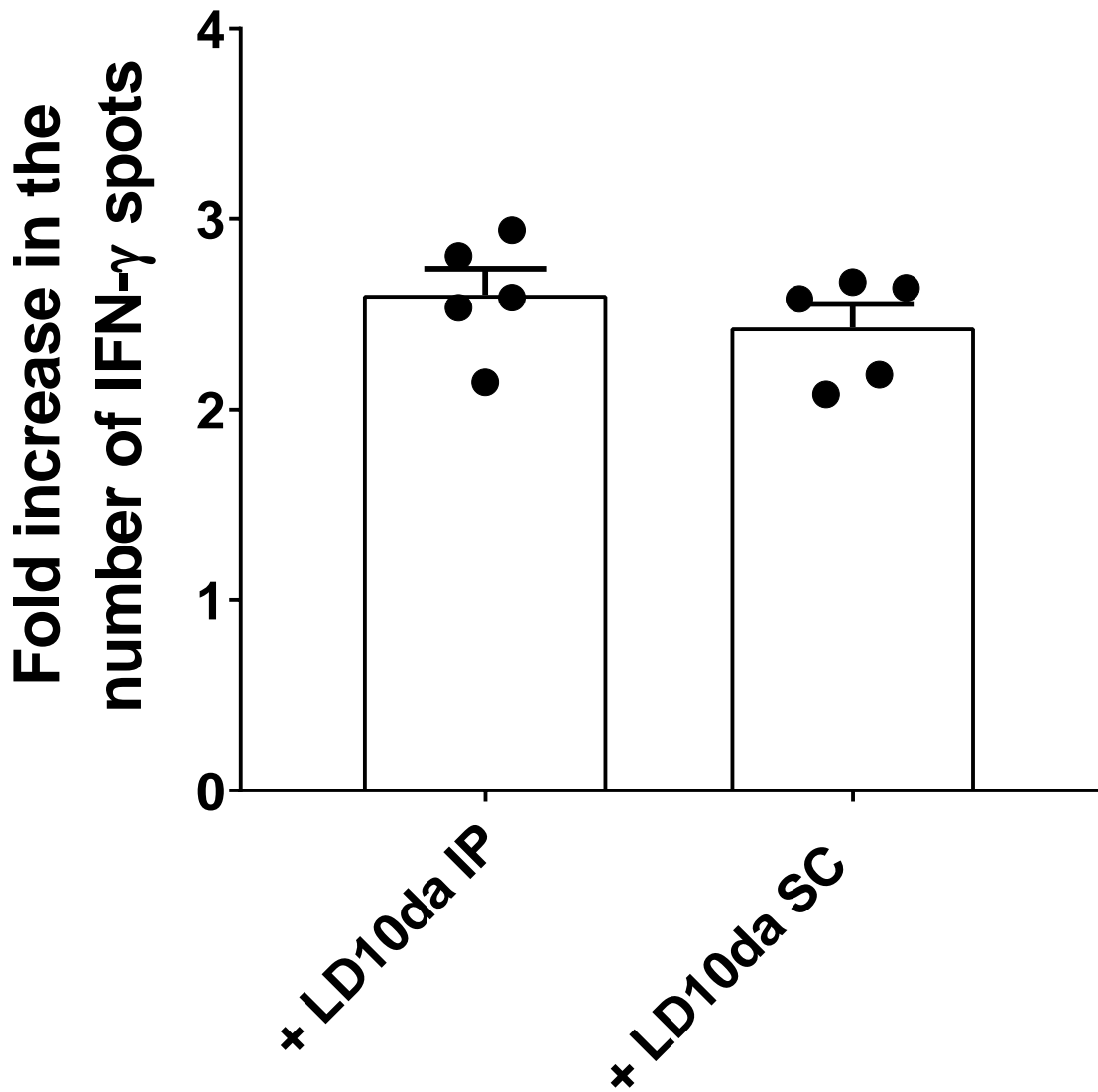

Supplement: Supplementary file 1 [file DataSheet1.PDF]
